# Development and evaluation of a venous computed tomography radiomics model to predict lymph node metastasis from non-small cell lung cancer

**DOI:** 10.1097/MD.0000000000020074

**Published:** 2020-05-01

**Authors:** Mengdi Cong, Haoyue Yao, Hui Liu, Liqiang Huang, Gaofeng Shi

**Affiliations:** a Department of Computed Tomography and Magnetic Resonance, Children's Hospital of Hebei Province; b Department of Computed Tomography and Magnetic Resonance, Hebei Medical University Fourth Affiliated Hospital and Hebei Provincial Tumor Hospital, Shijiazhuang, Hebei Province; c Cooperate Research Center, United Imaging Healthcare, Shanghai, China.

**Keywords:** forecasting, lymph nodes, non-small cell lung cancer, radiomics, tomography, X-ray computed

## Abstract

The objective of this study was to develop a venous computed tomography (CT)-based radiomics model to predict the lymph node metastasis (LNM) in patients with non-small cell lung cancer (NSCLC). A total of 411 consecutive patients with NSCLC underwent tumor resection and lymph node (LN) dissection from January 2018 to September 2018 in our hospital. A radiologist with 20 years of diagnostic experience retrospectively reviewed all CT scans and classified all visible LNs into LNM and non-LNM groups without the knowledge of pathological diagnosis. A logistic regression model (radiomics model) in classification of pathology-confirmed NSCLC patients with and without LNM was developed on radiomics features for NSCLC patients. A morphology model was also developed on qualitative morphology features in venous CT scans. A training group included 288 patients (99 with and 189 without LNM) and a validation group included 123 patients (42 and 81, respectively). The receiver operating characteristic curve was performed to discriminate LNM (+) from LNM (−) for CT-reported status, the morphology model and the radiomics model. The area under the curve value in LNM classification on the training group was significantly greater at 0.79 (95% confidence interval [CI]: 0.77–0.81) by use of the radiomics model (build by best 10 features in predicting LNM) compared with 0.51 by CT-reported LN status (*P* < .001) or 0.66 (95% CI: 0.64–0.68) by morphology model (build by tumor size and spiculation) (*P* < .001). Similarly, the area under the curve value on the validation group was 0.73 (95% CI: 0.70–0.76) by the radiomics model, compared with 0.52 or 0.63 (95% CI: 0.60–0.66) by the other 2 (both *P* < .001). A radiomics model shows excellent performance for predicting LNM in NSCLC patients. This predictive radiomics model may benefit patients to get better treatments such as an appropriate surgery.

## 1 Introduction

Lung cancer is a major cause of cancer-related death worldwide and is one of the most common malignant tumors in China.^[1]^ Early detection and staging of tumors are important for the management of lung cancer.^[2]^ The National Comprehensive Cancer Network guidelines (2018) state that surgery should be the first choice for early non-small cell lung cancer (NSCLC) patients, and radiation and chemotherapy should be the first choice for advanced NSCLC patients.^[3]^ The most common metastatic pathway of lung cancer is lymphatic metastasis. Lymph node metastasis (LNM) is an important factor to determine lung cancer staging, treatment plan, and prognosis. Preoperative understanding of tumor metastasis can provide valuable information for determining the need for adjuvant therapy and the adequacy of surgical excision, thus helping clinicians to make correct decisions.^[4]^ At present, there are many methods to evaluate whether NSCLC lymph nodes (LNs) have metastasis, and these methods include computed tomography (CT), positron emission tomography (PET)-CT, ultrasound-guided biopsy, thoracoscope, and so on.^[5,6]^ Pathology-related biopsy and thoracoscopy evaluate the LN staging better than radiological images, however, these would be invasive procedures for the patients. PET-CT is a noninvasive method which is used in cancer staging, and a growing number of multidisciplinary research teams of lung cancer prefer to use PET-CT for lymphatic metastasis evaluation.^[7,8]^ Many studies reviewed the diagnostic performance of PET-CT in LN staging of NSCLC. Noninvasive PET-CT is a relatively accurate imaging technique, which has high specificity for LNM in preoperative NSCLC patients. However, the misdiagnosis rate^[9]^ and false negative rate^[10]^ were higher in PET-CT diagnosis of LNM than the gold standard pathological results. In addition, PET-CT is unaffordable for most patients, especially in underdeveloped and developing countries, which also limits its large-scale clinical application.^[11]^ Therefore, a noninvasive and cost-effective evaluation tool is needed to predict whether there is LNM in primary lung cancer patient.

Recently, radiomics has received more and more attention from radiologists. It provides a noninvasive assessment for the characteristics of tumor tissue which has great prospects.^[12]^ Medical imaging has been routinely used for the diagnosis, staging, and treatment of tumors.^[13,14]^ Radiomics features were extracted from high-throughput medical images and logistic regression model which was built on the selected radiomics features was applied in various tumor managements. The application of radiomics depends on radiologists, who are required to firstly outline the regions of interest (ROI), and then calculate the quantitative texture features.^[15]^

Currently, radiomics has been reported to predict LNM of colorectal cancer, bladder cancer, breast cancer, and lung adenocarcinoma, and it has higher sensitivity and specificity than traditional methods of assessing LNM.^[16,17]^ Previous studies^[17,18]^ predicting LNM were all interested in lung adenocarcinoma, and the sample size was small. These studies suggested that it was reasonable to analyze the primary tumor itself and predict LNM. And our study was focused on patients with NSCLC, which was different from the previous researches. The pathological diagnosis of NSCLC needed to be confirmed by invasive procedures such as biopsy or surgery. We believed that the postsurgical diagnosis (eg, lung adenocarcinoma, squamous cell carcinoma, adenosquamous carcinoma, large cell carcinoma, and so on) could not be presurgical parameters since the radiologists could not make imaging diagnosis as exactly as the pathological finding on the thin-section CT scan. That was the reason for using all the types of NSCLC patients to develop the radiomics model in our study. Accurate clinical stage is very important for the treatment of NSCLC.

This study developed a CT-based radiomics model to predict the probability of LNM in NSCLC, and then to guide the clinical selection of appropriate surgical population.

## 2 Materials and methods

### 2.1 Patients

An institutional review board waiver was obtained for this retrospective study. Patients retrospectively met the following criteria from January 2018 to September 2018 in our hospital:

(1) Patients underwent radical resection of lung cancer with LN dissection.
(2) Postoperative pathology was NSCLC and LNM was reported.
(3) Patients had complete preoperative clinical data.

**Figure 1:**
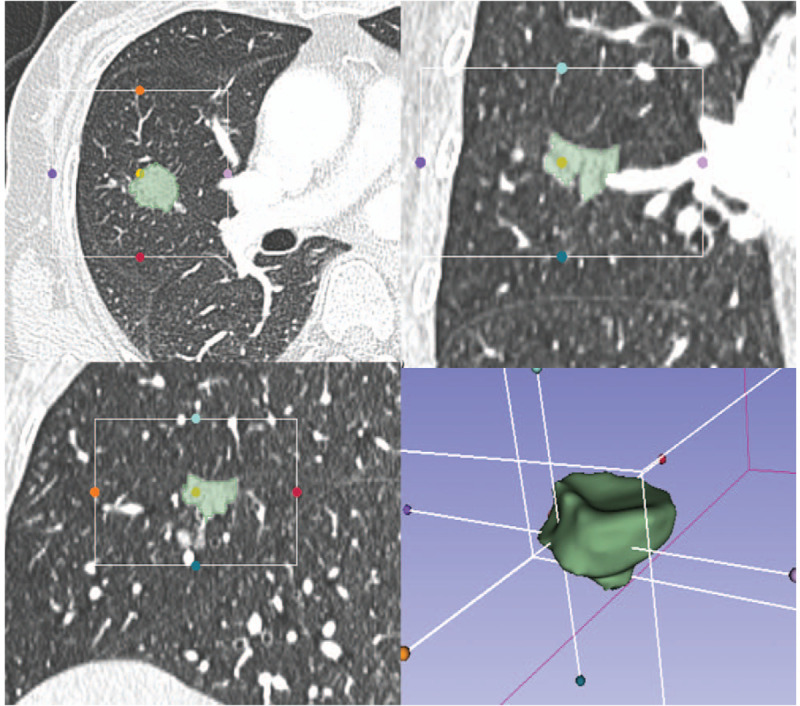
A representative CT region of interest segmentation diagram. CT = computed tomography.

Exclusion criteria:

(1) Patients had received radiotherapy and chemotherapy before surgery.
(2) Patients underwent CT examination more than 1 month before surgery.
(3) Patients did not have a thin film enhanced chest scan.
(4) Patients had distant metastasis. Patients’ selection process is shown in Figure 1.

A total of 411 patients were randomly divided into the training group and the validation group according to 7:3. Training set included 288 cases, where 99 of them had LNM and the 189 left were not. Another 123 cases were used as the independent validation set (42 of them had LNM and the 81 left were not). The clinical and histopathologic data of the patients were evaluated, including age, gender, smoking, drinking, family history of lung cancer, histological type (adenocarcinoma, squamous carcinoma, others) and LN status. This study had been reviewed by the ethics committee of our hospital.

LN status preoperatively was evaluated by a variety of standards. Benign (negative) LNs were defined as follows: calcification and fat in the LN. Malignant (positive) LNs were defined as follows: short axis of the LN ≥10 mm, necrosis in the LN.^[19]^ The LN status reported on CT was evaluated on preoperative CT by 2 radiologists (with 5 and 3 years of experience) without the knowledge of pathological diagnosis separately. If the 2 radiologists did not have the same opinion, the decision was made after consultation with a senior physician (20 years of experience in chest diagnosis). Tumor node metastasis (TNM) classification and preoperative clinical stages were based on the most recent (8th edition) international staging criteria published by the Union for International Cancer Control and the American Joint Committee on Cancer.^[20]^

### 2.2 CT image acquisition

All patients underwent enhanced chest spiral CT scanning. The screening machine was the second generation SOMATOM Definition Flash CT scanner of Siemens. The patient was examined in the supine position. The scan range was from the entrance of the chest to the lower lung (costophrenic angle), continuous screening with single breath at the end of inspiration.

Scanning settings: slice thickness, 1.0 mm; tube voltage, 120 kVp; tube current, 80 to 300 mAs. All images were displayed with standard lung (width, 1200 HU; level, –600 HU) and mediastinal (width, 350 HU; level, 40 HU) window settings. The contrast agent was injected into the elbow vein. The flow rate was 3 mL/s, the dose was 1.5 to 2 mL/kg, and the venous phase scan was delayed for 90 seconds. All images were exported in digital imaging and communications in medicine format for image feature extraction.

### 2.3 Quantitative and qualitative feature definition

The quantitative features were age, size (maximum tumor diameter). The qualitative features were gender, smoking status, drinking status, family history, location of tumor (upper location contains left and right upper lobes; middle-lower location means right middle lobe, left and right lower lobes), lobulation, spiculation, pleural indentation, vacuole sign, and CT-reported LN status.

### 2.4 Radiomics feature extraction and selection

**Figure 2:**
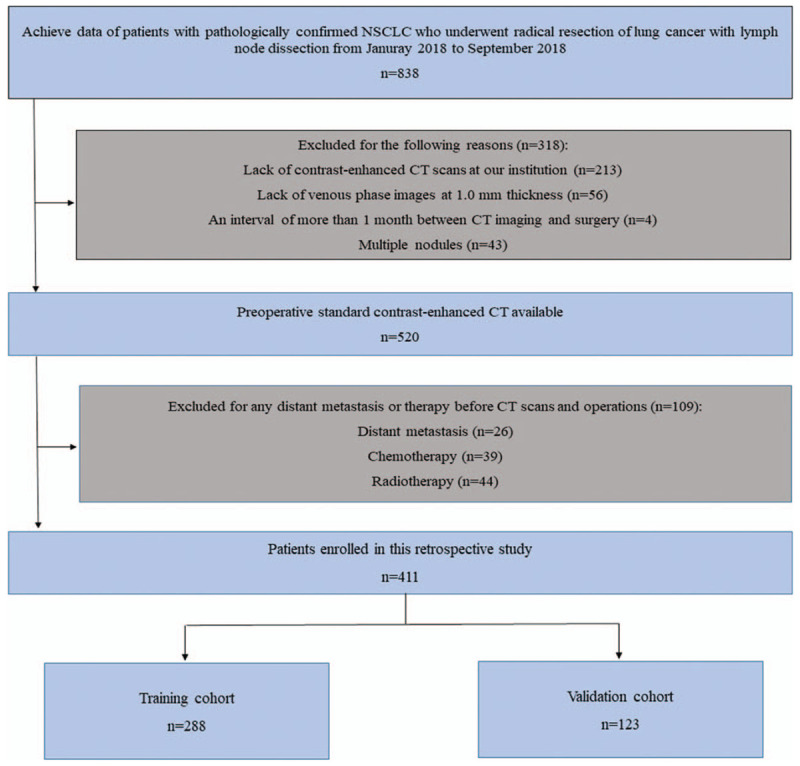
Recruitment pathway for patients in this study.

The ROI for the primary tumor was semi-automatically drawn by a 5-year radiologist using the segment editor (grow-cut ROI toolbox) of 3D slicer (www.slicer.org). The tumor ROI considered the bronchi, blood vessels, and vacuoles inside the tumors, excluding the normal lung tissue. A representative CT region of interest segmentation diagram is shown in Figure 2. Pyradiomics (version 2.0.0, https://github.com/Radiomics/pyradiomics) was used to extract features from lung lesions with settings as followed: bin size is 64, voxel shift is 1000, voxel size is 1 mm isotropic, filters include LoG (1.0, 2.0, 3.0, 4.0, 5.0) and wavelet (LLH, LHL, LHH, HLL, HLH, HHL, HHH, LLL).^[21]^ A total of 1229 radiomics features were extracted and grouped as First Order Statistics (19 features), Shape-based (26 features), Gray Level Cooccurrence Matrix (24 features), Gray Level Run Length Matrix (16 features), Gray Level Size Zone Matrix (16 features), Neighboring Gray Tone Difference Matrix (5 features), and Gray Level Dependence Matrix (14 features). By applying a Laplacian of Gaussian filter with 5 sigma levels (1.0, 2.0, 3.0, 4.0, 5.0), 421 features were obtained. And 688 wavelet features were extracted. Each numerical radiomics feature was normalized using the *z*-score method before input to the model.

Features selection is a process to further improve the robustness and accuracy of machine learning model by selecting mostly contributed features to the prediction results to reduce the overfitting. We applied the univariate feature selection method (analysis of variance) for each feature to test its *F*-value belonging to TNM(−) or TNM(+) groups. The best of 10 features (by soring *F*-value) were selected to be input to the model.

### 2.5 Model

We used logistic regression with least absolute shrinkage and selection operator (LASSO) constrain as the classifier for both qualitative CT morphology features and radiomics features. L1 norm in the LASSO regression model adds the absolute value of weighing coefficient term as penalty term in the lost function, and shrinks the less important feature's weighting coefficient to 0. The performance of the model was evaluated using receiver operating characteristic (ROC) curve analysis. The area under the ROC curve (AUC) was calculated for quantification. The accuracy, sensitivity, specificity, positive predictive value (PPV), and negative predictive value (NPV) were also calculated at a cut-off value of the radiomics score (LASSO output probability with range from 0 to 1, defined as the linear combining of selected features with their weighting) to evaluate the efficiency of the final models. The modeling and ROC performance evaluation were performed in a machine learning modeling toolbox (FeAture Explorer v0.2.2, https://github.com/salan668/FAE), which were implemented on the basis of Scikit-learn python package (https://scikit-learn.org).

### 2.6 Statistical analysis

Continuous variables were reported as mean ± standard deviation, while categorical variables were performed as counts. The differences of continuous variables were analyzed by paired *t* test including age and tumor diameter. Differences of categoric variables were analyzed by Chi-square test including sex, smoking, drinking, history, tumor location, lobulation, spiculation, pleural indentation, vacuole sign, and type of tumor.

ROC was used to evaluate the prediction performance for CT-reported LN status, morphological features and radiomics features on MedCalc (https://www.medcalc.org, version: 18.11.6). Delong test was used to test the statistical significance of the difference among the areas under ROC curves, and *P*-value of less than .01 was considered as significant.

## 3 Results

### 3.1 Patient characteristics

From January 2018 to September 2018, 411 consecutive patients met the inclusion criteria, including 204 males and 207 females, with averaged mean age of 59.62 (24–84). The characteristics of all patients are summarized in Table 1. Postoperative pathology confirmed the presence of LNM in 141 cases, with a metastatic rate of 34.3%. Tumor size was 2.80 ± 1.40 cm, including 144 cases with diameter greater than 3 cm and 267 cases with diameter less than 3 cm.

**Table 1:**
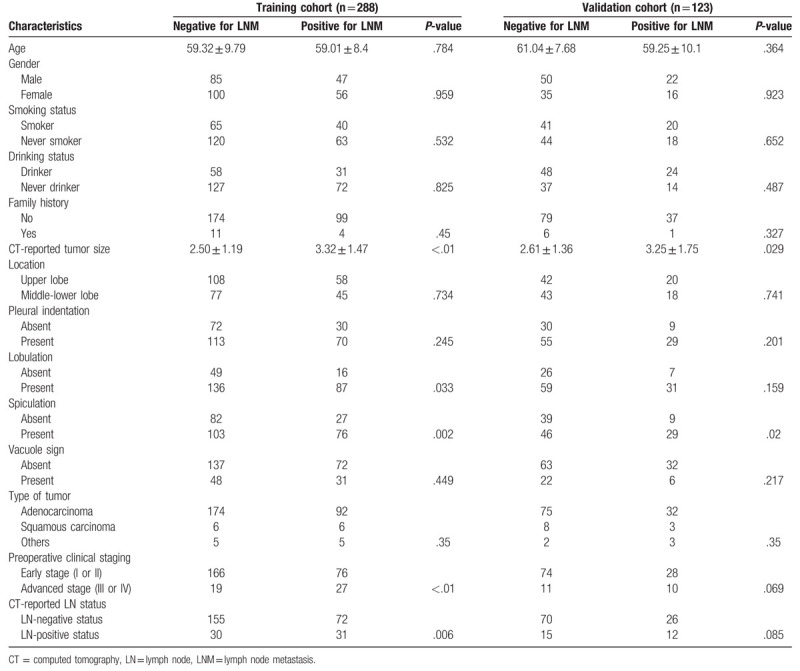
Characteristics of patients in the training and validation cohorts.

### 3.2 ROC analysis

**Figure 3:**
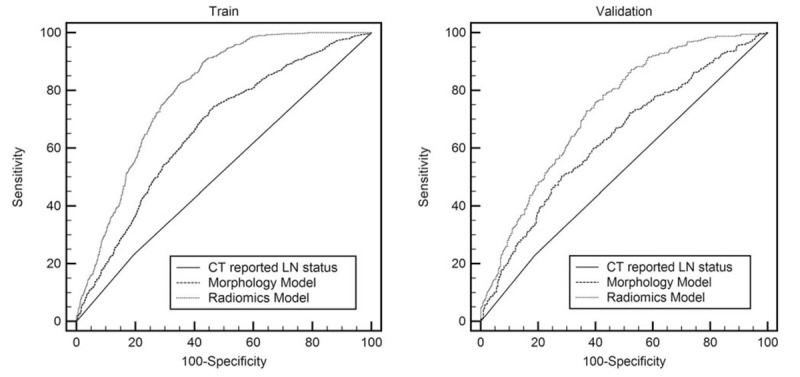
Comparison of ROC curves among the CT-reported LN status, morphology model and radiomics model respectively to prediction the lymph node metastasis in the train cohorts (A) and validation cohorts (B). CT = computed tomography, LN = lymph node, ROC = receiver operating characteristic.

The ROC curves comparison for CT reported LN status, morphology model and radiomics model were shown in Figure 3.

In the CT reported LN status, the AUC value in the training group is 0.51 (95% confidence interval [CI]: 0.49–0.53), and the AUC value of the validation group is 0.52 (95% CI: 0.48–0.55).

CT-reported tumor size and spiculation (*P* < .05) were chosen to build the morphology model. The AUC value of the model is 0.66 (95% CI: 0.64–0.68) and 0.63 (95% CI: 0.60–0.66), respectively in the training and validation group.

For the LASSO model using radiomics features, 10 features with highest AUC on the training data set were selected, where its feature weight is listed in Table 2. The AUC value of the training group is 0.79 (95% CI: 0.77–0.81), with an accuracy of 0.710, a sensitivity of 0.812, a specificity of 0.603, a PPV of 0.680, an NPV of 0.756. The AUC value of the validation group is 0.73 (95% CI: 0.70–0.76) for the radiomics model, with an accuracy of 0.725, a sensitivity of 0.778, a specificity of 0.672, a PPV of 0.704, an NPV of 0.752.

**Table 2:**
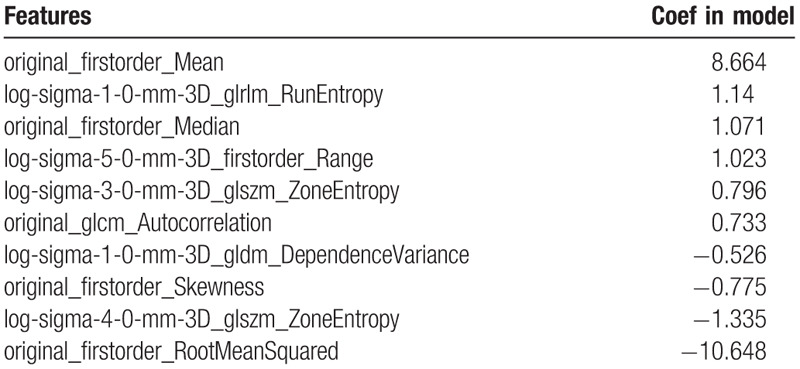
The coefficients of features in the model.

Delong test shows that the *P*-values for pairwise comparison between radiomics model, CT reported LN status and morphology model are all less than .0001. Therefore, there is significant difference between CT reported LN status, qualitative morphology model and radiomics model, where the radiomics model gives the best performance.

## 4 Discussion

The study used radiomics model to identify LNM in NSCLC. We used a radiomics model that contained the radiomics signature for prediction of LNM in patients with NSCLC before surgery. The radiomics model we established showed good identification ability in both the training cohort (AUC, 0.79) and the validation cohort (AUC, 0.73), and it was superior to the CT reported and morphological prediction model for LNM. We usually diagnose LNM by mediastinal LN enlargement, calcification, and so on. In our experiment, the AUC value of the CT reported LN status model was 0.51 and 0.52, respectively in the training and validation cohort, indicating a low prediction efficiency. This study showed that features such as tumor diameter and spiculation were of great significance in identifying LNM in NSCLC. Some studies had shown that tumor size was associated with LNM in NSCLC.^[22,23]^ As the diameter of the tumor increased, the proportion of LNM was rising, which was consistent with the results of this study. In our research, we demonstrated that tumor with spiculation was more likely to have LNM. We found that spiculation was a predictive feature of invasiveness and poor prognosis.^[18,24]^ We believed that the possibility of LNM was greater in tumors with spiculation. As a qualitative indicator, morphology had great subjectivity in the clinical work, and senior radiologists had obvious advantages of diagnosis. In our experiment, Radiomics features addressing the heterogeneity such as original first order mean and root mean squared had the greatest correlation. Mean represents the average pixel mean of an image. Skewness and entropy were also the features which could predict LNM for NSCLC. Skewness represents the asymmetry of the image histogram distribution. The larger the value is, the more asymmetric the histogram (lesion) distribution of the image will be; the smaller the value is, the more symmetrical the histogram distribution will be. Entropy refers to the uniformity of pixel value distribution in the image histogram. The higher the entropy is, the more evenly the image pixel value is distributed. They were reported as predictors of LN metastasis in other malignant tumors.^[25,26]^ The efficacy of these radiomics features in predicting LNM was significantly higher than that of CT-reported LN status model and morphology model.

Our research showed that LNM in some patients could not be seen on CT images, which was in compliance with the previous studies.^[17,18]^ In recent years, radiomics has been widely used in medical imaging and clinical. Yan Zhong et al analyzed 492 patients and predicted occult mediastinal LNM in lung adenocarcinoma.^[17]^ But their study only predicted LNM in patients who were not clinically detected instead of analyzing the false positive patients. In our study, patients who were clinically diagnosed with Nx were also included, and some of them had actually pathological results of N0. In addition, the nonenhanced scan images of lung cancer were selected for ROI drawing in this study. In our study, enhanced images of NSCLC patients were selected for ROI outline. Systematic mediastinal lymphadenectomy was an important part of surgical treatment of early stage NSCLC. LN dissection of patients with NSCLC had a greater trauma and had a great impact on their recovery.^[27]^ Therefore, in order to avoid these effects, the clinical requirements for high-precision and noninvasive methods to predict LNM of NSCLC were increasingly high. We needed to assess whether the patient had LNM accurately to improve the sensitivity and to reduce the false positive rate. Previous studies had reported that the pathological type and histological grade of patients were predictors of early LNM in NSCLC.^[17,22,23]^ These data could only be collected after the surgery, so they cannot be used as a guide for LN dissection. More and more early stage NSCLC was found and received surgical treatment. Surgical resection was the first choice for NSCLC patients with clinical stage I, II, and part III. Systematic mediastinal lymphadenectomy was still controversial in these patients.

This study had limitations. First of all, this was a retrospective study, which would lead to migration. Second, this study only used the enhanced CT image. Because the thickness of the nonenhanced lung CT image was 5 mm, which would lead to the reduction of some image features, we only selected the thin-slice contrast-enhanced scanning image.

Through statistical analysis of CT morphology, radiology and clinical data of NSCLC, we established a radiomics model by using LASSO method, which could predict the probability of LNM of NSCLC and guide clinical decision better.

In conclusion, we proposed a radiomics model established by logistic regression to predict LNM in NSCLC, and this model could guide clinical decision better. The performance of the radiomics model was much better than the traditional morphology model.

## Author contributions

**Conceptualization:** Gaofeng Shi.

**Data curation:** Mengdi Cong, Haoyue Yao.

**Formal analysis:** Haoyue Yao.

**Funding acquisition:** Gaofeng Shi.

**Investigation:** Mengdi Cong.

**Methodology:** Mengdi Cong, Gaofeng Shi.

**Software:** Mengdi Cong, Hui Liu, Liqiang Huang.

**Writing – original draft:** Mengdi Cong, Hui Liu.

**Writing – review & editing:** Gaofeng Shi.

Mengdi Cong orcid: 0000-0002-1371-2301.
